# Operation Rooms, past and Present
1An address delivered before a professional and lay audience on the occasion of the opening of the modernised operation room at the Bristol Royal Infirmary on September 30th, 1898.


**Published:** 1898-12

**Authors:** William MacCormac

**Affiliations:** President of the Royal College of Surgeons of England; Surgeon in Ordinary to H.R.H. the Prince of Wales; Consulting Surgeon to St. Thomas's Hospital, London


### OPERATION ROOMS, PAST AND PRESENT.[1]

BY

Sir William MacCormac, Bart., K.C.V.O.,

*President of the Royal College of Surgeons of England; Surgeon in Ordinary to H.R.H. the Prince of Wales; Consulting Surgeon to St. Thomas's Hospital, London.*

---

It was with much pleasure that I accepted the compliment you have paid me to-day by inviting me to open your new operating theatre, which the wise generosity of the subscribers has given to your Infirmary. In his interesting statement of the details of its construction your architect, Mr. W. H. Thorp, has afforded us an account of what a modern operating theatre should be. It is worthy of your ancient Infirmary, which, founded in 1735, has, I am told, the prestige of being one of the earliest in the country out of the metropolis. In an operating theatre are typified or symbolised the advances of modern surgery, which have been so amazing during the past quarter of a century.

I can remember the conditions under which operations were performed at a time which to many present may appear ancient

[1] An address delivered before a professional and lay audience on the occasion of the opening of the modernised operation room at the Bristol Royal Infirmary on September 30th, 1898.

history. It was a time when anæsthesia, which revolutionised surgery, had but very recently come into general employment. I can remember the almost complete neglect, in many places at any rate, of sanitary conditions in the theatre itself: the absence of any sufficient regard to surgical cleanliness, or often indeed ordinary cleanliness, both in the case of the surgeon who operated and of the person of the patient upon whom the operation was to be performed. Any place was considered good enough for an operating room. There were generally sinks which communicated with defective drains, floors and benches innocent of the scrubbing-brush or soap and water; marine sponges were commonly used, and these proved a fertile source of infection from being imperfectly cleansed and made use of over and over again. I have known theatres that were placed in immediate proximity to the dead-house, to which they too often proved the portal.

Oiled lint or warm-water dressings were applied to the wounds, or dirty *charpie* stuffed into them, as was the practice in France. Immediate union of the wound was never thought of or attempted abroad. Sometimes no dressing was applied, that discharges might have free vent. But presently foul-smelling pus flowed from the wounds; they were swollen, painful, and inflamed. The patient had high fever, and often and often and often erysipelas would set in, or pyæmia, or some other form of septic poisoning, and the patients too frequently died. The surgeon would give his hands a perfunctory washing, perhaps not even do that. I have often seen surgeons proceed from one operation to another without cleaning their hands at all. There would be no cleansing of the patient's body. The operator usually donned an old black coat, copiously stained with the blood of previous patients, and of which the older and dirtier it was he usually felt the more proud; and he would be assisted by those who were as little careful in these respects as himself. What wonder then if such neglect of what now we know to be all-essential precautions should be followed by disastrous consequences!

I well remember one of my earliest operations, soon after I qualified as a surgeon. A young ruddy countryman, in typical good health, had his little finger crushed. I amputated it—perhaps the smallest amputation one could perform, except the little toe—and he died soon after of pyæmia. I was horrified and distressed at so dreadful an issue to so simple an operation. Occurrences like this were by no means uncommon. In every hospital traumatic fever, so called, which we now know to be a form of septic contamination, erysipelas, prolonged suppuration, or fatal pyæmia, hospital gangrene even, were disastrously frequent.

I can recollect my experience during the Franco-German war. It was heart-breaking. It is just twenty-eight years ago since I entered the town of Sedan on my way to join MacMahon and his army as a volunteer ambulance surgeon. But next day the army began to join us; it was in full retreat, and at four a.m. on that eventful September 1st, 1870, began the battle of Sedan. A hospital of 500 beds was hastily extemporised, the day before the battle, in a soldiers' barracks on the ramparts of Sedan. It was dirty enough, as you may suppose, having been but a day or two before evacuated by the troops. Into this streamed wounded men from early morning of the 1st September, 1870. The soldiers' camp-beds were soon filled, and other poor fellows lay along the passage-ways and on the staircases. There was more than enough for us to do, dressing the slighter injuries, performing operations on those more severely injured. Our antiseptic material was scanty, and, I fear, ineffectively employed. The overcrowding was great, and the sanitary arrangements bad; and what was the result? These young soldiers, healthy and in the prime of life, battered a little no doubt by the hardships of the campaign, with long marching and insufficient food, died almost like flies of their wounds, died from simple flesh wounds, died for the most part after gunshot fractures, died from secondary hemorrhage after the ligature of a great artery, and in every instance from what was then called blood-poisoning in some form or another. I did what I could, but the numbers were too many for me. I will tell you of one incident which illustrates the state of things at that time. A French *Intendant General* visited the hospital and ordered the windows to be closed. "You will kill your patients," he said, "with *courants*

*d' air*," and this on a fine, warm September day! The *Intendant General* was an officer, non-medical himself, who had absolute control in the French army of everything medical.

The Grand Hotel in Paris during the siege of the city was turned into a hospital, and during that time I believe it to be absolutely true that of the many hundreds of amputations there performed all alike proved fatal from septic poisoning.

Our campaign in Egypt in 1882 was the first English one in which antiseptics were generally used in strict fashion. It showed how all this might be changed, for during that campaign, and for the first time I believe in the history of military surgery, there was not amongst the wounded men a single case of septic infection or of erysipelas. A very marvellous transformation!

Professor Reyher, of St. Petersburg,—who, like your Greig Smith, died too young,—with Professor Bergmann, then of Dorpat, now of the Royal Clinic in Berlin, astonished the surgical world by the results of their antiseptic treatment of penetrating gunshot injuries of the knee inflicted during the Russo-Turkish war of 1875. Before this the almost invariable treatment of such injuries was amputation; of the slighter cases which were treated conservatively during the American war more than 60 per cent. died. These surgeons were able through antiseptic methods to save, not only the life and limb of the individual, but even to preserve in many instances the perfect use of the joint.

In the recent brilliant campaign on the Nile, where the military medical arrangements were of the most complete and perfect nature, I hear that some of the wounded men complained of being neglected, because, forsooth, their wounds were not dressed every day. One of the achievements of the modern surgical methods is to render these daily dressings wholly unnecessary.

I need not say when or where, but I have many times seen great fat white maggots crawling about a putrescent wound; and a most loathsome sight it was. And it is a matter of history that this was often witnessed during the Crimean War, where, it is interesting to read, chloroform was first employed in military surgery.

Now all this has changed, and through the beneficent influences of anæsthesia and of antiseptics we have been able to realise the immense achievements of modern surgery. I have said these are typified to a great extent in a surgical operating theatre as we now understand one. It should be of moderate size, so that it can be quickly and readily cleansed; apart, if possible, from the rest of the building, so that it may be open all round, and have a thorough ventilation of air. The walls should be of impermeable material—glass or marble—and perfectly plain, no angles anywhere for the lodgment of dust; the floor of the theatre of mosaic or marble, and the auditorium, or place for spectators, the same. All sinks, slabs for holding dressings, or trap wash-basins should be supported on cantilever brackets, so that a clear space is all round. The sinks should be of porcelain, plentifully supplied with hot and cold water, draining into an open culvert in the floor, and be provided with pedal taps. All complicated drain pipes and traps should be avoided.

The operating table should be glass and iron, and the furniture generally, instrument cases, &c., of the same materials.

Sterilised water should be provided by a suitable filter, and an abundant supply of sterile saline solution for washing out cavities. There should be sterilising apparatus for the instruments; and all dressings, towels, bandages, anything in short used for the patient, should be sterilised also. In St. Thomas's Hospital a "Theatre Sister" has charge of the arrangements in the operating theatre, and of the instruments, and of dressings most especially; and the plan has worked admirably.

The daylight should be from the north; and there should be an abundance of hanging and movable electric lamps for night use.

The air introduced into the theatre should be filtered, and its temperature regulated according to the season. If abundantly forced in, it finds its own way out; and an apparatus for this purpose keeps the atmosphere pure, the temperature regulated, and does away with any supposed necessity for a glass screen interposed in some places between the operating area and the spectators.

The surgeon, his assistants, the nurses also, should be clad in sterilised over-alls, and forceps used in place of fingers to hand sponges and dressings. The surgeon's hands must be thoroughly cleansed. I need not detail how this is done, but I think the best way of keeping them aseptic is by frequent washing in some weak antiseptic solution during the progress of the operation. As much care must be taken by the assistants as by the surgeon himself; and I cannot myself see that wearing gloves, which must be very inconvenient, or coating the hands with a layer of impermeable material, which is hardly less so, afford any proportionate advantage. There are many ways of rendering the area of the surface of operation aseptic but I shall not trouble you with further details.

All these requirements are capable of fulfilment here, and are worthy of the town in which have worked such distinguished men as Richard Smith, Augustin Prichard, and Greig Smith, as well as many others. Richard Smith was surgeon of this Infirmary for forty-seven years. He founded the museum, and was in his time, 1796 to 1843, a very able and distinguished surgeon. Augustin Prichard is but lately dead, but whilst he flourished he was the best known surgeon in the West of England. The son of this distinguished father serves the Hospital in a no less distinguished manner now. Not only Bristol, but England—surgery indeed everywhere—deplores the premature loss of Greig Smith. He was a great surgeon and a modest man. His indomitable energy wore out the machine at the early age of 43. But his work on *Abdominal Surgery* lives after him, and will continue to live; and this theatre is an admirable memorial of him. There can be no doubt that the able followers of these illustrious predecessors will do full justice to their enhanced opportunities.

A very distinguishing feature of the new surgery is its decentralisation. Fifty years ago, or less, all the more important surgical cases, when the means of the individual could afford it, would frequently go to London for advice and operation. This practice obtains no longer, except to a much limited extent. Provincial surgeons and provincial surgery are so good, there can be no longer any reason for it. The new order may, however, have its inconveniences in regard to

London surgeons; but these must be allowed to have small weight when compared with the great advantages the change affords for the community at large. But, after all, the loss is more apparent than real, for the number of operations performed has so enormously increased. A large number, and it will be an ever-increasing number, of operations, are provided by the physicians from the medical wards. Improved methods of diagnosis, the almost complete safety with which many classes of operation can now be dealt with, and the inestimable benefits of anæsthesia, have increased tenfold the possibilities of the operating theatre. In St. Thomas's Hospital they have increased from about eight hundred to nearly three thousand per annum in the last ten years.

Year by year medicine is advancing. It will advance in this community—it has already very notably advanced—and it will advance also in other centres and countries; and I am sure I may most heartily congratulate this Infirmary, and the surgeons who belong to it, on the possession of so admirable a means for the development of their art which has been, with so excellent a generosity, placed at their disposal.

I have only, in conclusion, to offer you my best acknowledgments for the patience with which you have listened to this very fragmentary discourse.

